# Feasibility of cancer genetic counselling and screening in Cameroon: perceived benefits and barriers

**DOI:** 10.3332/ecancer.2023.1588

**Published:** 2023-08-14

**Authors:** Berthe Sabine Esson Mapoko, Kenn Chi Ndi, Lionel Tabola, Vanessa Mouaye, Pelagie Douanla, Nasser Nsangou, Glenda Nkeng, Carmen Vanvolkenburgh, Bonaventure Dzekem, Dezheng Huo, Paul Ndom, Olufunmilayo Olopade

**Affiliations:** 1 Department of Internal Medicine and Specialties, Faculty of Medicine and Biomedical Sciences, The University of Yaoundé I, Yaoundé 99322, Cameroon; 2 National Cancer Control Committee, Yaoundé 99322, Cameroon; 3 Center for Global Health, University of Chicago Medical Center, Chicago, IL 60637, USA

**Keywords:** cancer genetic counselling, screening, Cameroon, benefits, barriers

## Abstract

Because there was no genetic testing service in Cameroon, we assessed the acceptance, perceived benefits and barriers and willingness to pay for genetic cancer screening in Cameroon amongst patients with cancers. We carried out a hospital-based, cross-sectional study on adult cancer patients at the Yaoundé General Hospital and the non-Governmental Organisation Solidarity Chemotherapy between February 1, 2021, and December 31, 2021. This was a convenience sampling that included all consenting patients. Qualitative and quantitative data were analysed by Epi info version 7 and SPSS version 20. Our study included 160 (87.5% females) cancer patients, whose ages ranged from 20 to 82 years, with a mean of 49.9 ± 13.0 years. Only 11.9% had undergone some form of genetic counselling or information sessions, and most found this to be helpful in terms of increased knowledge and prevention strategies (13, 68.4%). Almost all participants (156, 97.5%) stated they will like their relatives to undergo genetic counselling. Of these, 151 (94.4%) expressed their desire for their relatives to discuss their cancer risk with a specialist. Perceived benefits of genetic testing included cancer prevention (108, 67.5%) and motivation of self-examination (81, 50.6%). Prominent possible barriers included the cost (129, 80.6%), unavailability of equipment (49, 30.6%) and anticipated anxiety (40, 25.0%). However, a majority of the participants (156, 97.5%) were willing to test for genetic mutations. One hundred and thirty-five (84.4%) participants were willing to pay for genetic testing, with the majority of them (71.8%) ready to pay between $16.7 and $100. Almost all of the participants expressed their willingness to receive cancer genetic counselling and testing but the cost became the main barrier. This pilot study will serve as a guide to the processes of establishing a cancer risk assessment clinic in Cameroon.

## Introduction

Given that cancer is a heterogeneous disease and that individuals have varying risks depending on environmental and genetic factors, risk-based screening strategies are on the rise [1]. Several studies have demonstrated that cancer can be initiated from either hereditary factors, environmental factors or a combination of both [2–8]. It has also been reported that genetic variables may interact with environmental factors and modifying factors (such as poor diet, smoking, physical activity and other lifestyle factors) to affect the cancer risk in human populations [1]. Hereditary cancer accounts for about 5%–10% of all malignancies [9]. Identifying these patients is of utmost importance because unlike patients with sporadic cancers they require special, long-term care due to risk of more severe disease forms, and increased risk of family members developing cancer [9–12]. There is a wide knowledge gap in cancer genetics among black Africans, with data showing that only 0.329% of cancer publications globally were on Africa, and only 0.016% was on cancer genetics from Africa [9]. Most of these studies were from the North African populations [9–16]. Hence, there is a need for a concerted effort to address the gaps in cancer genetics in Sub-Saharan African populations [9, 17, 18]. Adedokun *et al* [19] showed that there is a high proportion (15.8%) of mutations in BRCA1/2 among patients with symptomatic breast cancer in Cameroon and Uganda. In the same way Zheng *et al* [20] and al showed a high proportion (14.7%) of mutations in BRCA1/2 among patients with symptomatic breast cancer in Nigeria. Cameroon is a middle-income country of Central Africa with 26,545,864 of total population [21]. The World Health Organisation in 2020 estimated 20,745 new cases of cancer diagnosed in Cameroon, with 13,199 deaths [21]. Cancer is still underdiagnosed in Cameroon owing to the difficulty to access healthcare services due to financial and geographical limitations [22, 23]. There is no universal health coverage plan in Cameroon; with most of its population having little to no knowledge on health insurance. A recent study revealed that only 4.4% of Cameroonians had health insurance [22, 23]. This shows the difficulty to access healthcare services coupled with the shortage of health personnel [23, 24]. About 80% of the patients have a late arrival to the hospital, with advanced stages at diagnosis and a treatment dropout rate of 20.0% due to inability to pay for medical care [23, 25]. This late arrival is also due to inadequate diagnosis by general practitioners leading to time lost before coming for the specialist consultation, and beliefs, fears, cultural factors, and ignorance [23, 25]. There are currently neither cancer genetic counselling services, nor any cancer genetic testing laboratories in Cameroon. Genetic counselling and testing services are presented as potential solutions to help achieve the Cameroon’s National Strategic Plan for Cancer Prevention and Control (NSPCPC), 2020–2024 [26]. In Cameroon, there are 200 different tribes that speak many African languages and dialects. The French and English languages both have official status, with the majority of Cameroonians being French-speaking [26]. This ethnic and language diversity has implications in the implementation of genetic counselling services. A recent study on the implications on public of cancer genetic services identified the following as potential areas to be addressed: 1) prioritisation of infrastructures, 2) need for translational research, 3) information dissemination to potential users, 4) training programs for specialised personnel, and 5) engaging political stakeholders and the public [27]. As preliminaries to this, patients need to be prepared for the establishment of genetic counselling and screening services in our setting. Genetic counselling is the process of helping people understand and adapt to the medical, psychological, and familial implications of genetic contributions to disease [28]. Counselling is defined as a purposeful relationship between two people, the first one is the client and the second one is the counsellor or the researcher herself, who approach a mutually defined problem with mutual consideration of each of them to the end that the troubled one or less mature is aided to a self-determined resolution of his problem [29–32]. Screening and genetic counselling program was introduced in 1946 [33]. Counselling for early detection and management of various menstrual disorders can improve the quality of life of the complaining women, mitigate their symptoms and minimise the debilitating health problems [34]. Using the family illness history, genetic counselling estimates the objective risk of developing cancer. Lower risk offers reassurance while high risk allows patients and their families to make informed decisions about their health, present and future. Once a genetic mutation has been identified in a patient, testing of at-risk relatives can identify those family members who also have the familial mutation. This will subsequently lead to increased surveillance to identify and diagnose a cancer earlier [35]. This study was conducted prior to the implementation of cancer genetic counselling and screening services in Cameroon, and aimed to assess knowledge about cancer, acceptance of cancer genetic counselling, perceived benefits of cancer genetic counselling, perceived barriers to testing and willingness to get tested and to pay for genetic cancer screening amongst patients with cancer.

## Patients and methods

We carried out a cross-sectional study in Yaoundé, Cameroon, between February 1, 2021, and December 31, 2021. Patients were recruited from the Yaoundé General Hospital; a tertiary reference hospital that serves as one of the three major cancer treatment centres in Cameroon, and the Solidarity Chemotherapy (Solidarité Chimiothérapie or SOCHIMIO), a non-governmental organisation (NGO) that has worked over the years to make treatment more affordable for patients. We included all adult cancer patients, followed at the NGO Solidarity Chemotherapy and the Yaoundé General Hospital who consented to participate. Trained research associates approached the patients during their outpatient consultation sessions, explained the study to them and obtained informed consent. Patients were then interviewed using a semi-structured questionnaire (see Supplementary Data). The questionnaire was developed based on extensive literature search on the current subject and was assessed by a variety of experts in the field of oncology and genetics. The questions were organised in the five sections. Our outcomes of interest included: i) knowledge of patients about cancer, ii) their acceptance of cancer genetic counselling, iii) the perceived benefits of cancer genetic counselling for risk assessment in patients' relatives, iv) their perceived barriers to testing, and v) their willingness to get tested for gene mutations and to pay for genetic cancer screening. The questionnaires were formulated in English and translated to French, the dominant language in Cameroon. English speaking participants were given the questionnaires in English while French speaking participants were given that in French. The interviewers were bilingual and related with the participants based on their dominant language. Descriptive statistics such as mean and SDs was used to summarise quantitative variables and frequencies and proportions for qualitative variables. Data were analysed with Epi info version 7 and SPSS version 20. The confidentiality of study participants was ensured. Several steps were taken to protect their anonymity and identity. Coded study ID were used instead of names on all data collected. Only the investigator, co-investigators, research associates and analysts had access to the raw data. Ethical approval was obtained from the National Human Health Research Ethical Committee reference N^0^2021/12/1424/CE/CNERSH/SP.

## Results

We interviewed 160 participants with cancers. The majority (75.0%) of patients had breast cancer, followed by ovarian cancer (12%), prostate cancer (6%), melanoma, gastric cancer and colorectal cancer (Table 1). There were 140 (87.5%) females and 20 (12.5%) males, with ages ranging from 20 to 82 years, with a mean of 49.9 ± 13.0 years. The most common age group was the 40–49 years-old group followed by the 50–59 years group as shown in Table 1.

A majority of the participants (103, 64.4%) were married. Most participants (71.2%) had at least a secondary education, while 3 (2.3%) had no formal education at all. Sixty-eight (42.5%) participants had a family history of cancer. Of these, 32 (47.1%) had at least a first degree relative with cancer, 34 (50%) had a second degree and 2 (209%) had a third degree. It is worth noting that 53 (77.9%) of these 68 participants with a positive cancer history had just 1 relative with cancer, 12 (17.6%) had 2 relatives and 3 (4.4%) had up to 3 relatives with cancer.

### Knowledge of patients about cancer

The majority of participants (57.5%) had no idea of the causes of their cancer, while 24.4% said that the cancer came from genetic/hereditary factors, and 18.1% said that the cancer come from lifestyle and environmental factors.

Only 19 participants (12%) had previous genetic counselling or education.

**Figure 1.**
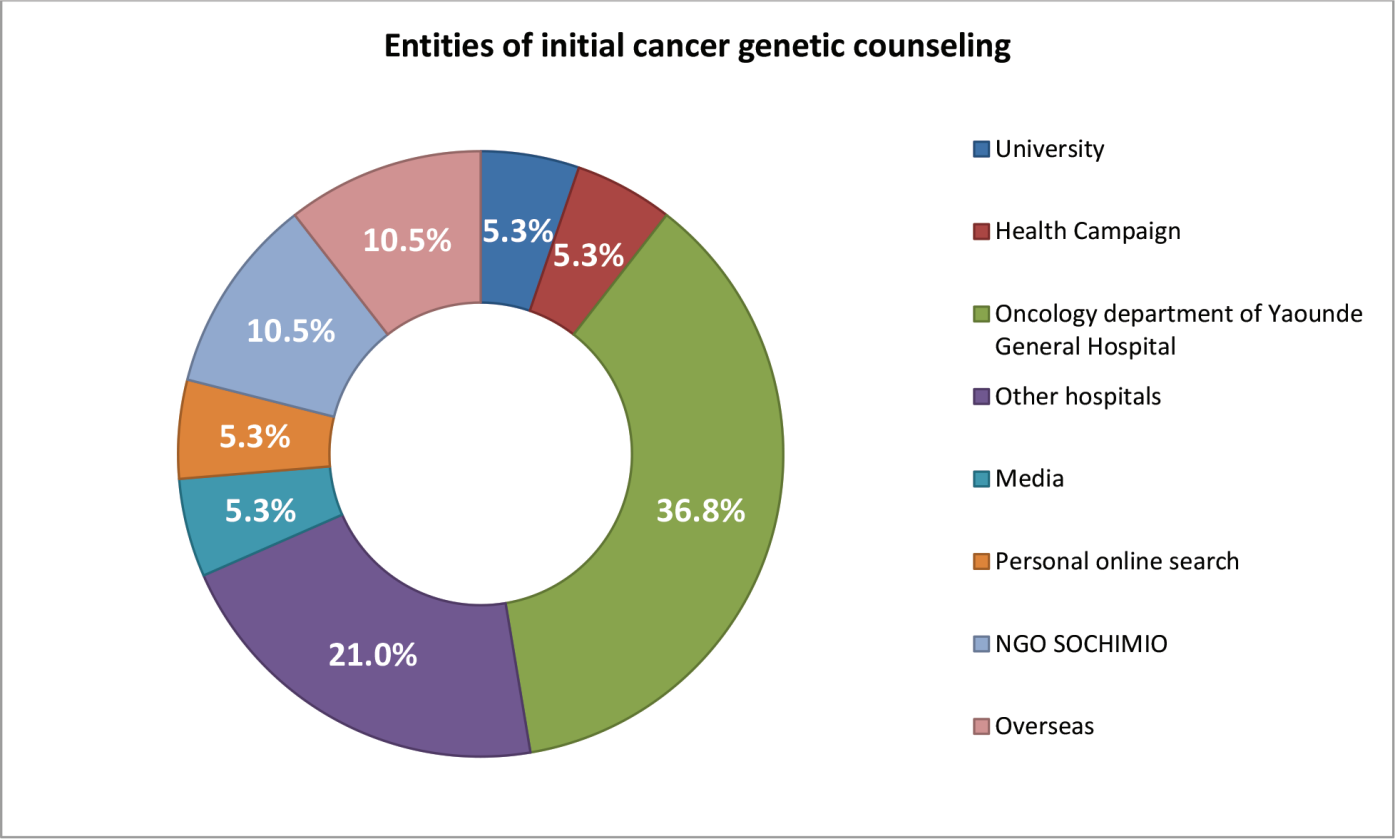
Entities that provided initial cancer genetics counselling/education and proportion of patients who received it.

When we asked those who had received some sort of education or counselling on cancer genetics about their sources, most [7] had received this information from the Oncology Department of the Yaoundé General Hospital, four from other hospitals, two each from overseas hospitals and the NGO SOCHIMIO, and one each from personal online research, the media, health campaigns and from the University (Figure 1). Most of these participants found this information to be useful, mostly in terms of increased information and prevention strategies (13, 68.4%).

### Acceptance of cancer genetic counselling

Most (144, 90%) participants stated that they were willing to undergo cancer genetic counselling if it was offered with 106 (66.3%) being concerned about their relatives getting cancer. However, only 1 (0.6%) denied it while 15 participants (9.4%) were unsure. Concerns ranged from the fear of transferring the cancer-associated genes to their children and relatives, the fact that their relatives could die from cancer, the financial burden of the disease and the suffering and stress associated with the disease.

Almost all participants (156, 97.5%) stated they would like their relatives to undergo genetic counselling. Of these, 151 (94.4%) expressed their desire for their relatives to discuss their cancer risk with a specialist. Further, 148 (92.5%) participants said they will like their relatives to get genetic testing.

### Perceived benefits of cancer genetic counselling

**Figure 2.**
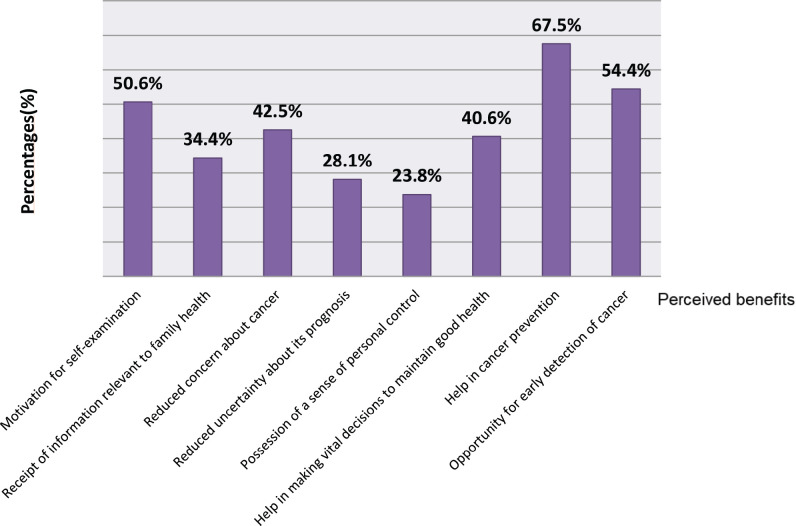
Perceived benefits for patients and relatives undergoing genetic counselling (part 1).

Perceived benefits of genetic testing included help in cancer prevention (108, 67.5%) and motivation of self-examination (81, 50.6%). Other perceived benefits included opportunity for early detection of cancer (54.4%), making vital decisions to maintain good health (40.6%), reduced concern about cancer (42.5%), receipt of information relevant to family health (34.4%), reduced uncertainty about cancer’s prognosis (28.1%) and possession of a sense of personal control (23.8%) (Figure 2).

**Figure 3.**
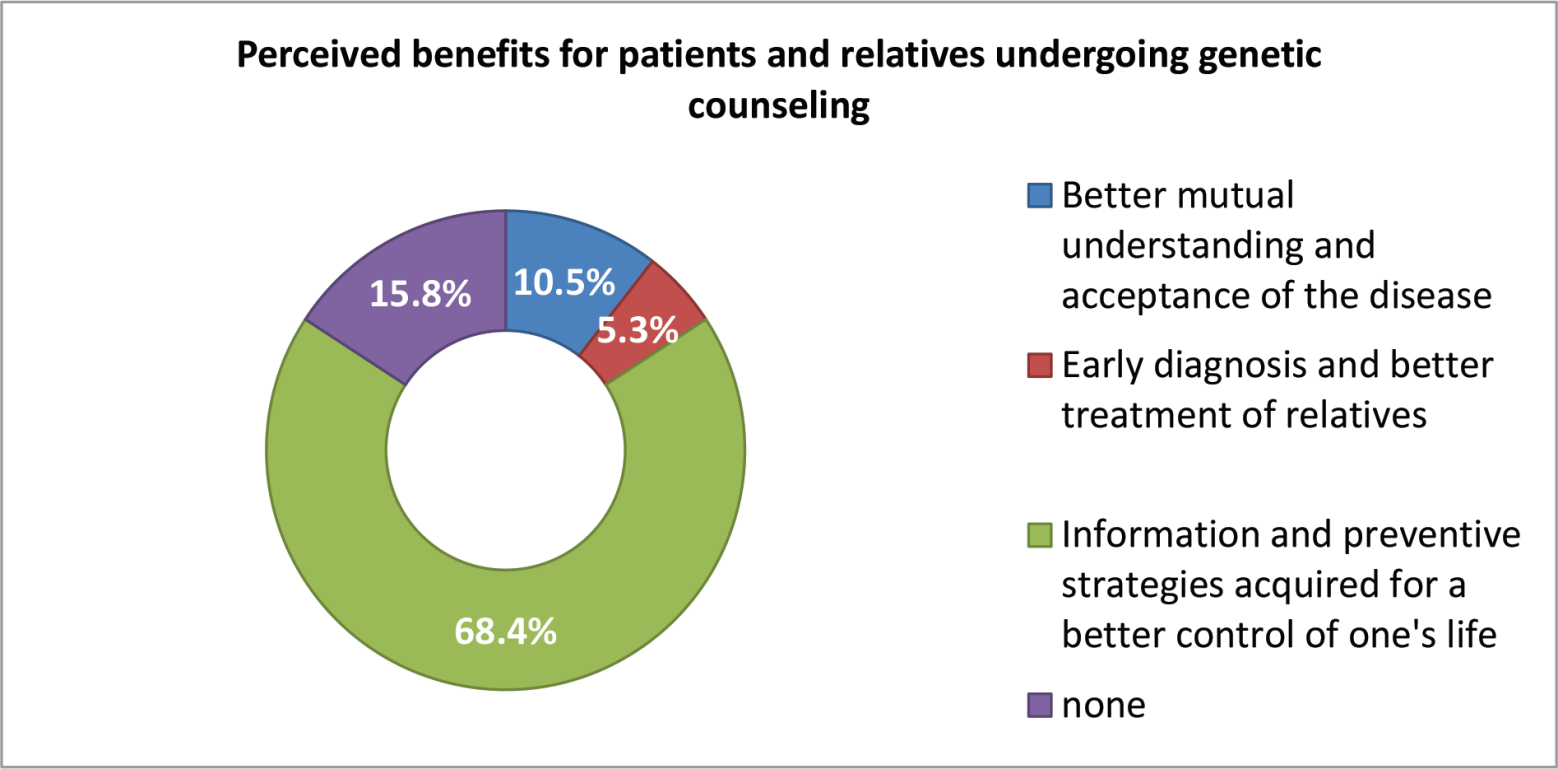
Perceived benefits for patients and relatives undergoing genetic counselling (part 2).

These benefits were also perceived including better mutual understanding and acceptance of the disease, early diagnosis and better treatment and information and preventive strategies acquired for a better control of one’s life perceived with respect to testing both participants and their relatives (Figure 3).

### Perceived barriers of cancer genetic testing

**Figure 4.**
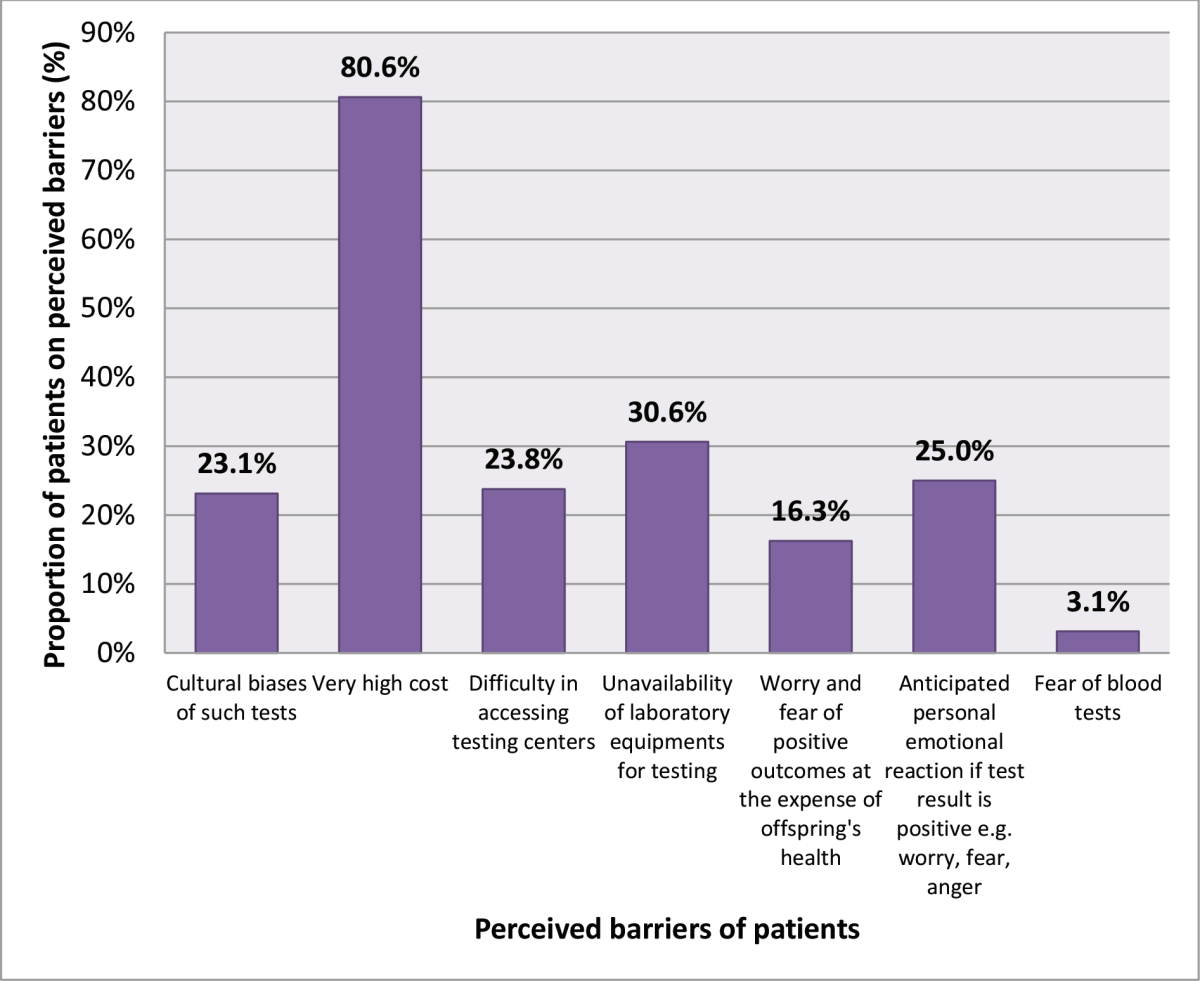
Perceived barriers for cancer genetic testing.

Prominent possible barriers included the cost (129, 80.6%), unavailability of equipment specially the genetic test itself (49, 30.6%) and anticipated anxiety (40, 25.0%). Other barriers were cultural biases of such tests (23.1%), difficulty in accessing testing centres (23.8%), worry and fear of positive outcomes at the expense of offspring’s health (16.3%) and fear of blood tests (3.1%) (Figure 4).

### Willingness to undergo testing and to pay for genetic test

**Figure 5.**
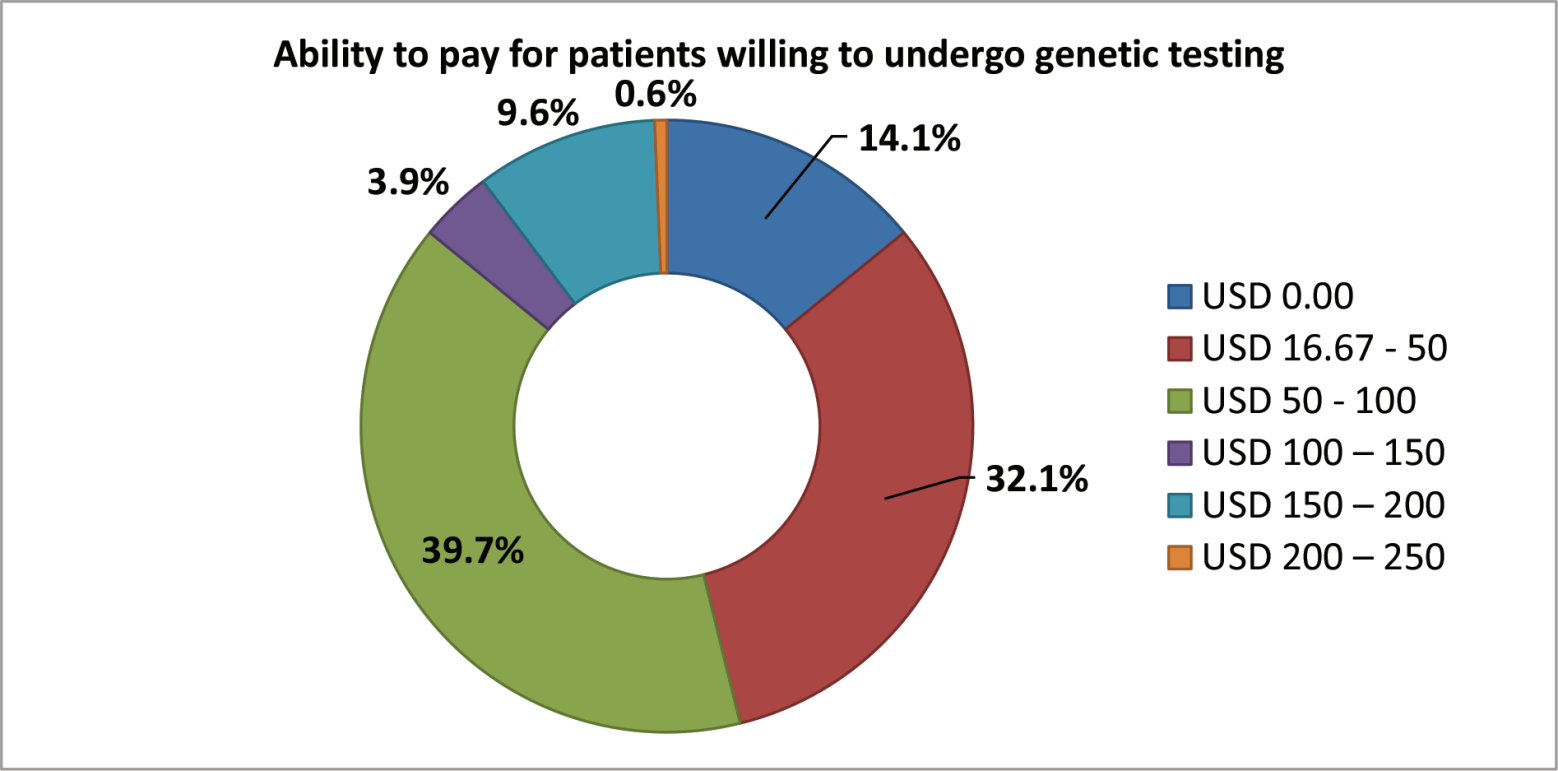
The ability to pay in patients willing to undergo genetic testing.

Almost all participants (156, 97.5%) were willing to test for genetic mutations. One hundred and thirty-five (84.4%) participants were willing to pay for genetic testing, with the majority of them (71.8%) ready to pay between $16.7 and $100. Figure 5 shows the range of patients willing to pay for genetic testing. All those who refused to pay for genetic testing (*n* = 25) stated it was expensive given their current financial burden of cancer. We then asked them if they would be ready to undergo genetic testing if this was free and most (155, 96.9%) answered yes.

One hundred and thirty-six (85.0%) said they will be ready to discuss the results of their genetic testing with their relatives. Those who refused to share this said they did not want to bother them or cause them to panic.

## Discussion

The present study which aimed to assess the feasibility of cancer genetic counselling and screening in Cameroon showed a tenth of our participants had heard about cancer genetics. Almost all participants stated they will like their relatives to undergo genetic counselling. They had some perceived benefits of genetic testing not withstanding some major prominent possible barriers. Albeit, almost all participants were willing to get tested for genetic mutations with most being ready to pay.

More than half of the study participants had no idea as to the causes of their cancer, similar to the findings of previous studies done in Nigeria [36, 37]. This may be due to similar public health concerns faced by these countries, where the level of health education is still low. Moreover, there was a gross disparity between the proportion of those who had a family history and those who thought cancer could be hereditary. This may reflect the lack of cancer awareness and education in our setting [13–15, 38–46]. The Cameroon Cancer Control Committee through its NSPCPC is currently addressing this issue through mass education campaigns [26]. Only a tenth of our participants had heard about cancer genetics, probably due to the fact that there is no cancer genetic counselling service in the country. In a study of a representative sample of the Canadian population, 59% of the population said to have no knowledge of genetic counselling and thought that counselling may help in disease prevention, similar to the participants in our study [47]. The disparity in the proportion of those unaware of genetic counselling; 59% in Canada to 88% in Cameroon may be due to the availability of genetic services in their setting unlike ours. This is contrasting to the findings of a study in the United States of America where almost 70% declared to have received appropriate genetic testing and counselling [48]. This may be due to the availability of genetic services and the fact that most genetic tests are covered by national insurance plans in some developed countries [49]. Moreover, most of those who had some knowledge on cancer genetics had gotten this information from the Yaoundé General Hospital. At this hospital, the head nurse counsels all patients who come for their first time about cancer risk; including lifestyle and family predisposition. It could be plausible to assume that an intensification of cancer education will improve patients’ understanding of the genetic risk of cancer and may improve their consideration for genetic counselling and testing as has been shown in studies in other underserving community [50, 51]. The 19 participants who had received cancer genetic information said it was helpful in terms of helping them adopt preventive strategies for the development of a second cancer and for the prevention of cancer in their relatives.

Most of the participants in this study were willing to undergo genetic counselling and were concerned about their relatives developing cancer. This is the logical line of thought for most of these participants, given that our interviewers were the first persons to present information on genetic screening and counselling to them. These findings are similar to those obtained from the study carried out by Adejumo *et al* [37, 52] as most of those who underwent genetic counselling had similar concerns to those in our study and all accepted to undergo testing. Most patients further went ahead to mention that they will like their relatives to discuss their cancer risk with a specialist. This finding is similar to that in a British study where participants who were undergoing genetic counselling for the first time said they will be more comfortable to be seen by an expert as this was associated with receiving full information [53]. The perception that either they or the doctor were actively able to do something about their situation helped to relieve feelings of vulnerability. In the same way, our patients will be relieved if a specialist talks with their relatives about their cancer risk. This may be associated to their feeling of being responsible for ‘bringing cancer into the family’, and counselling with its associated benefits of prevention and early detection, could be seen as a salvation from this perceived ‘curse’.

The main perceived barriers to genetic cancer genetic counselling and testing were the cost, inaccessibility and anxiety as well as cultural biases. These findings are similar to those uncovered by Adejumo *et al* [54] in Nigeria where 80% of their participants said the cost of genetic counselling was the most significant barrier to genetic testing and counselling. Difficulty to access testing sites (55.3%), unavailability of tests (38.3%), anticipated anxiety (38.3%) and cultural biases (23.4%) were other remarkable barriers to testing in their study. Zhong *et al* [55] equally had similar findings in their study on the opportunities and barriers for genetic service delivery in Kenya. These corroborations may be due to the many similarities in ethnic origins, similar economic challenges and cultural similarities. A study done in the United Kingdom on the hindrances to cancer genetic testing among ethnic minorities also found sociocultural beliefs as a barrier and proposed the introduction of culturally sensitive provider and counselling initiatives, and by enabling of patient self-referral as facilitators to the access of these services in these minority groups [56]. A similar study done in the United States of America found that cancer genetic counselling programs led by accepted and trusted individuals from the community will reduce these sociocultural barriers [51]. Therefore, it will be necessary for us to train personnel in different communities from the diverse ethnic groups of Cameroon, who will then provide cancer genetic education to these populations. Further study is required to understand the particular ethnic barriers across the diverse groups so as to better understand how to address this in planning the establishment of cancer genetic services.

Most of our participants were willing to undergo genetic testing, most (71.8%) ready to pay between $16.7 and $100. In comparison, the Nigeria study reported in 54% are willing to pay $22–$65 [52]. So, a genetic service with a patient out of pocket cost around $50 might be sustainable from patients’ perspective. It implies the need to have insurance or government to cover the remaining cost. The actual cost of genetic counselling and testing is higher than $200, which only covers cost of the testing in developed countries, although most of these are covered by national insurance coverage [49, 57]. Similarly, most were ready to undergo the test if it was free of charge. This is in line with the challenges found in other African studies of which the cost stood out as one of the most remarkable limitation [54, 55]. Most of our participants (85.0%) said they will be ready to discuss the results of their genetic testing with their relatives. Those who refused to share this said they did not want to bother them or cause them to panic. Some may have refused because there is still a stigma attached to cancer in less developed settings and people may be unwilling to be tagged with bringing bad luck to the family.

## Conclusion

This pilot study could serve as a guide to establishing a cancer genetic counselling and risk assessment testing clinic in Cameroon and potentially in other low- and middle-income countries.

Almost all of the study participants expressed their willingness to receive cancer genetic counselling. The cost of genetic testing represented the greatest barrier for testing in populations with low income. To address this, we are collaborating with researchers at the University of Chicago to apply for funds for free tests to initiate the cancer genetic service, but there should be sustainable resources allocated to genetic testing by the government or other organisations. There is also a plan in place to start a genetics laboratory in Cameroon, which is still in its early stages. It may also be helpful to talk about on ways to raise awareness in Cameroonian populations when it comes to cancer, but also genetic testing and counselling; forms of education and awareness could be discussed.

## Author contributions

**Conception and design:** Berthe Sabine Esson Mapoko, Kenn Chi Ndi, Lionel Tabola, Pelagie Douanla, Nasser Nsangou, Glenda Nkeng, Vanessa Mouaye, Carmen Vanvolkenburgh, Bonaventure Dzekem, Dezheng Huo, Paul Ndom, Olufunmilayo Olopade.

**Administrative support:** Bonaventure Dzekem, Dezheng Huo, Paul Ndom, Olufunmilayo Olopade.

**Provision of study materials or patients:** Paul Ndom.

**Collection and assembly of data:** Berthe Sabine Esson Mapoko, Kenn Chi Ndi, Lionel Tabola, Pelagie Douanla, Nasser Nsangou, Glenda Nkeng, Vanessa Mouaye, Carmen Vanvolkenburgh.

**Data analysis and interpretation:** Berthe Sabine Esson Mapoko, Kenn Chi Ndi, Carmen Vanvolkenburgh.

**Manuscript writing:** Berthe Sabine Esson Mapoko, Kenn Chi Ndi, Carmen Vanvolkenburgh.

**Final approval of manuscript:** All authors.

**Accountable for all aspects of the work:** All authors.

## Conflicts of interest

The other authors declare no conflicts of interest.

## Funding

The study is partially funded by National Cancer Institute of the National Institutes of Health (R01CA228198-03S1, Dezheng Huo).

## Figures and Tables

**Table:** 

	Causes	Yes	No
1.	I have no idea		
2.	Heredity/family history		
3.	Childlessness		
4.	Not breastfeeding well		
5.	Prolonged use of contraceptive pills		
6.	Obesity		
7.	Early menarche		
8.	Late menopause		
9.	Large breast		
10.	Unhealthy/fatty diet/highly processed food		
11.	Tight bras		
12.	Induced abortions		
13.	Alcohol consumption		
14.	Smoking		
15.	Antiperspirant		
16.	Stress		
17.	Previous history of breast cancer		
18.	**Others**		

**Figure:**
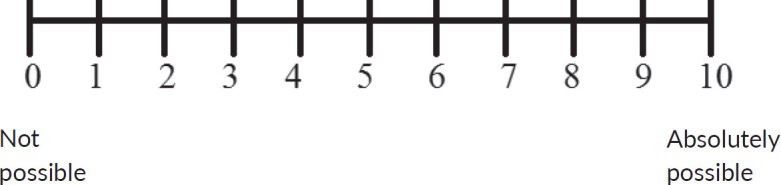


**Figure:**
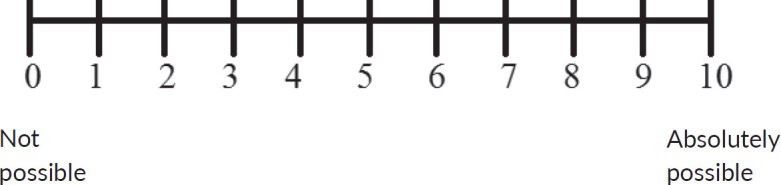


**Figure:**
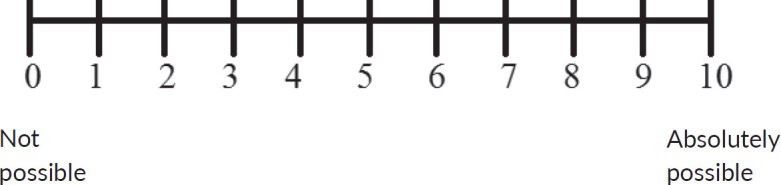


**Figure:**
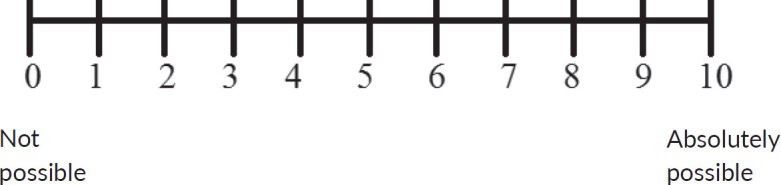


**Table:** 

	Treatment	Yes	No
1.	Chemotherapy		
2.	Surgery (lumpectomy)		
3.	Surgery (unilateral mastectomy)		
4.	Surgery (bilateral mastectomy)		
5.	Surgery (oophorectomy)		
6.	Radiotherapy		
7.	Any other (specify please)		

**Table:** 

S/N	Benefits	Yes	No
1.	Motivate self-examination		
2.	Receipt of information for family/being able to help family and children		
3.	Reduce concern about cancer		
4.	Reduce uncertainty		
5.	Provide a sense of personal control		
6.	Help plan for the future		
7.	Help make important life decisions		
8.	Help with cancer prevention		
9.	Opportunity for early detection of breast cancer		
10.	Other benefits		

**Table:** 

	Likely barriers	Yes	No
1.	Cultural perception of such test		
2.	Cost		
3.	Access to the testing centre		
4.	Availability of equipment for testing		
5.	Anticipated increased worry about offspring/relatives if test result is positive		
6.	Anticipated personal emotional reaction if test result is positive e.g., worry, fear, anger		
7.	Worry that others would find out		
8.	Time		
9.	Not wanting blood taken		
10.	Lack of interest		
11.	Worry about increased risk		
12.	Worry about discomfort		
13.	Other barriers		

**Table:** 

	Reasons for not wanting to test for mutations	Yes	No
1.	No need knowing if I have the genes		
2.	I don’t want to be labelled as bringing bad luck to the family		
3.	I will be worried unnecessarily if I test positive		
4.	I do not want blood draw		
5.	The test could cause discomfort		
6.	Other reasons		

**Table:** 

Relative	Yes	Relative	Yes	Relative	Yes	Relative	Yes	Relative	Yes
Father		Brother(s)		Daughter(s)		Aunt(s)		Grandmother	
Mother		Sister(s)		Son(s)		Uncle(s)		Grandfather	
Stepparent		Halfsibling(s)		Stepson/daughter		Cousin(s)		Spouse	

**Table 1.** Sociodemographic characteristics of the participants.

Characteristic	Number of patients	%
Age, years		
20–29	5	3.1
30–39	30	18.8
40–49	48	30.0
50–59	41	25.6
60–69	24	15.0
70–79	10	6.3
>80	2	1.3
Marital status		
Married	103	64.4
Single	34	21.3
Widowed	19	11.9
Divorced	4	2.5
Level of education		
None	3	2.3
Elementary	35	26.5
Secondary	51	38.6
University	41	31.1
Doctoral	2	1.5
Religion		
Christian	132	82.5
Muslim	24	15.0
Prefer not to answer	4	2.5
Type of cancer		
Breast	120	75
Ovarian	19	12
Prostate	9	6
Gastric	5	3
Melanoma	5	3
Colorectal	2	1
Family history of cancer		
Yes	68	42.5
No	92	57.5
1 relative affected	53	77.9
2 relatives affected	12	17.6
>3 relatives affected	3	4.4

## Supplementary Data

### Questionnaire

**Opinions of patients with genetic-associated cancers on genetic counselling and testing relating to cancer risks in their relatives: a study of Nigeria and Cameroon**

Dear participants,

Good day to you. You are invited to participate in a research study which focuses on exploring the opinions of patients with breast cancer on genetics and genetic counselling in respect of identifying risks of developing breast cancer in their relatives. The findings will be used to facilitate system reforms for the control of breast cancer.

I assure you that your responses to this questionnaire will be kept confidential. Your names and other identifiers are not required on the questionnaire. This is one of the ways of promoting anonymity of the responses. There are no risks or harm in participating and your participation is voluntary and you are free to withdraw from the study if you so decide at any time without any penalties. You are free to ask any question as you progress with the study.

Thank you.

Prof Paul Ndom

#### Section A: Socio-demographics

1. Age (as at last birthday) _______________________

2. Gender 1. Male □ 2. Female □

3. Marital status 1. Single □ 2. Married □ 3. Separated/Divorced □ 4. Widowed □

4. Religion 1. Christianity □ 2. Islam □ 3. Traditional religion □ 4. Others______

5. Ethnicity 1. Yoruba □ 2. Ibo □ 3. Hausa □ 4. Other_______

6. Highest educational qualification 1. Elementary □ 2. Secondary □ 3. Diploma/NCE □ 4. Bachelor’s degree □ 5. Master’s degree □ 6. PhD □

7. Occupation _____________________________________________

8. Monthly income __________________________________________

***Family history of cancer***

9. Has any member of your family had any type of cancer? 1. Yes □ 2. No □

10. If yes to question 9, who was the person? _____________________________________

11. How old was the person when the cancer was diagnosed? ___________________________

12. What type of cancer was diagnosed? ___________________________________________

*(Repeat questions 10–12 if there are more than 1 relative with cancer)*

#### Section B: Opinion of patient on the cause of their breast cancer

*(Tick Yes or No and give answers in your own word where required)*

13. When was the first time you were told you had breast cancer? _______ (In years or months)

14. What was the first symptom you noticed? ______________________________

15. When did you first noticed the symptom? ________________ (In years or months)

16. What is your understanding of your diagnosis of breast cancer?

____________________________________________________________________________________________________________________________________

____________________________________________________________________________________________________________________________________

17. In your own opinion, what do you think caused your breast cancer?

#### Section C: Willingness of patients to discuss breast cancer risk with their relatives

*(Circle the number picked by the patient on a scale of 0 – Not possible to 10 – absolutely possible)*

18. Risk of you developing breast cancer again (*What is the risk of recurrence of breast cancer?)*

19. Lifetime risk of any of my children developing breast cancer

20. Lifetime risk of any of my siblings developing breast cancer

21. Lifetime risk of any of my parent developing breast cancer

22. Have you ever discussed your breast cancer with your siblings?

1. Yes, I told all my siblings □

2. Yes, but not with all my siblings □

3. No, not at all □

23. If you discussed with your siblings, what made you do so?

____________________________________________________________________________________________________________________________________

24. If you discussed with only some of your siblings, what made you do so?

____________________________________________________________________________________________________________________________________

25. If you did not discuss with any of your siblings, what made you do so?

____________________________________________________________________________________________________________________________________

____________________________________________________________________________________________________________________________________

26. Have you ever discussed your breast cancer with your children?

1. Yes, I told all my children □

2. Yes, but not with all my children □

3. No, not at all □

27. If you discussed with your children, what made you do so?

____________________________________________________________________________________________________________________________________

28. If you discussed with only some of your children, what made you do so?

____________________________________________________________________________________________________________________________________

29. If you did not discuss with any of your children, what made you do so?

____________________________________________________________________________________________________________________________________

30. Have you ever discussed your breast cancer with your parent(s)?

1. Yes, I told both my parents □

2. Yes, but only one of my parents □

3. No, not at all □

31. If you discussed with both your parents, what made you do so?

____________________________________________________________________________________________________________________________________

32. If you discussed with only one of your parents, what made you do so?

____________________________________________________________________________________________________________________________________

33. If you did not discuss with any of your parents, what made you do so?

____________________________________________________________________________________________________________________________________

34. Have you ever discussed your breast cancer with any other relative(s)? 1. Yes □ 2. No □

35. If you discussed with other relative(s) who are the relatives?

____________________________________________________________________________________________________________________________________

36. If you discussed with other relative(s), what made you do so?

____________________________________________________________________________________________________________________________________

37. What treatment have you received since you started attending clinic at the hospital?

#### Section D: Perceived benefits of cancer genetic counselling on risk assessment of patients’ relatives

38. Have you ever had cancer genetic counselling before? 1. Yes □ 2. No □

39. If yes to question 38, where did you receive it? ____________________________________

40. If yes to question 38, when did you receive it? ____________________________________

41. If yes to question 38, what are the benefits of the counselling that you had?

____________________________________________________________________________________________________________________________________

____________________________________________________________________________________________________________________________________

42. If you have never had cancer genetic counselling, will you want to be counselled?

1. Yes □ 2. No □

43. Has any of your relatives had genetic counselling at the surgical outpatient clinic (SOP) where you see your doctor?

1. Yes □ 2. No □

44.If yes to question 43, which of your relatives had genetic counselling at SOP? (List as many relatives as possible) ___________________, ____________________, ________________

45. Do you have any concern about your relatives getting cancer?

1. Yes □ 2. No □

46. If yes to question 45, what concerns do you have?

____________________________________________________________________________________________________________________________________

____________________________________________________________________________________________________________________________________

47. Will you like your relatives to have cancer genetic counselling?

1. Yes □ 2. No □

48. Will you like your relatives to discuss their cancer risk with a specialist?

1. Yes □ 2. No □

49. Will you like your relatives to have cancer genetic testing?

1. Yes □ 2. No □

50. What benefits are available to your relatives if they undergo cancer genetics and risk assessment?

51. What are the likely barriers to genetic testing?

#### Section E: Intention of patients with breast cancer on genetic testing for mutations

52. Are you willing to test for genetic mutations for breast cancer? 1. Yes □ 2. No □

53. If no to question 47, why would you not want to test for genetic mutations?

54. If yes to question 47, are you willing to pay for genetic testing? 1. Yes □2. No □

In USA, genetic testing of this type cost $250 (N90,000), in South Africa R1500–13,400

(N44,175–394,6300), in UK between £1,500–2,000 (N647,625–1,295,250)

55. How much will you be willing to pay? 1. N10,000–N30,000□ 2. N40,000–N60,000□ 3. N70,000–N90,000□ 4. N100,000–N120,000□ 5. N130,000–N150,000□ 6. Others ______________________________(Specify)

56. If not willing to pay for genetic testing, what are your reasons? _____________________________

____________________________________________________________________________________________________________________________________

57. If it is free and the test is available now, would you like to be scheduled for testing?

1. Yes □ 2. No □

58. Will you like to discuss the result of your genetic test with your relatives? 1. Yes □ 2. No □

59. Who among your relatives will you like to discuss your genetic result with? (Choose from the list, as many as you will like to discuss with)

60. If no to question 58 give reasons _______________________________________________________________________

#### Section F: Personal history

***Your cancer health history*** □

61. Have you had (or do you currently have) cancer? 1. Yes □ 2. No □

62. Have you ever had colon polyps identified on colonoscopy or sigmoidoscopy?

1. Yes □ 2. No □ 3. I’m not sure □

63. Have you ever had a bone marrow transplant or blood transfusion? 1. Yes □ 2. No □

64. Have you ever had a genetic test for hereditary cancer risk? 1. Yes □ 2. No □

***Family tree***

65. Are you adopted, or do you have no health information about one, or both sides of your biological family members? 1. Yes □ 2. No □

66. Do you have any children? 1. Yes □ 2. No □

67. Do you have any siblings? 1. Yes □ 2. No □

68. Tell us about your parents and grandparents. Estimates are okay.

1. Mother’s name (optional): ____________________________ Mother’s age: _________

2. Father’s name (optional): ____________________________ Father’s age: _________

3. Maternal grandmother’s name (optional): ___________ Maternal grandmother’s age: _____

4. Maternal grandfather’s name (optional): _____________ Maternal grandfather’s age: _____

5. Paternal grandmother’s name (optional): _____________ Paternal grandmother’s age: _____

6. Paternal grandfather’s name (optional): _______________ Paternal grandfather’s age: _____

69. Does your mother have any siblings? 1. Yes □ 2. No □ 3. I’m not sure □

70. Does your father have any siblings? 1. Yes □ 2. No □ 3. I’m not sure □

71. Has anyone had a genetic test for hereditary cancer risk? 1. Yes □ 2. No □ 3. I’m not sure □

72. Is there anything else related to your personal or family history that you would like to share?

______________________________________________________________________________

**Thank you for your time!**
