# Erratum: Feasibility of cancer genetic counselling and screening in Cameroon: perceived benefits and barriers

**DOI:** 10.3332/ecancer.2023.1604

**Published:** 2023-09-25

**Authors:** Berthe Sabine Esson Mapoko, Kenn Chi Ndi, Lionel Tabola, Vanessa Mouaye, Pelagie Douanla, Nasser Nsangou, Glenda Nkeng, Carmen Vanvolkenburgh, Bonaventure Dzekem, Dezheng Huo, Paul Ndom, Olufunmilayo Olopade

**Affiliations:** 1 Department of Internal Medicine and Specialties, Faculty of Medicine and Biomedical Sciences, The University of Yaoundé I, Yaoundé 99322, Cameroon; 2 National Cancer Control Committee, Yaoundé 99322, Cameroon; 3 Center for Global Health, University of Chicago Medical Center, Chicago, IL 60637, USA

The author list for this article was incomplete - the correct author list should be:

**Berthe Sabine Esson Mapoko^1,2^, Kenn Chi Ndi^1^, Lionel Tabola^1^, Esther Dina Bell^3^, Vanessa Mouaye^2^, Pelagie Douanla^1^, Nasser Nsangou^1^, Glenda Nkeng^1^, Carmen Vanvolkenburgh^4^, Bonaventure Dzekem^4^, Prisca Adejumo^5^, Olutosin Awolude^6^, Toyin Aniagwu^7^, Dezheng Huo^4^, Paul Ndom^1,2^ and Olufunmilayo Olopade^3^**

^1^Department of Internal Medicine and Specialties, Faculty of Medicine and Biomedical Sciences, The University of Yaoundé I, Yaoundé 99322, Cameroon

^2^National Cancer Control Committee, Yaoundé 99322, Cameroon

^3^Department of Clinical Sciences, Faculty of Medicine and Pharmaceutical Sciences, University of Douala, Douala 99322, Cameroon

^4^Center for Global Health, University of Chicago Medical Center, Chicago, IL 60637, USA

^5^Department of Nursing, College of Medicine, University of Ibadan, Ibadan 200284, Nigeria

^6^Department of Obstetrics and Gynaecology, University College Hospital, Ibadan 200284, Nigeria

^7^School of Occupational Health Nursing, University College Hospital, Ibadan 200212, Nigeria

The acknowledgments section should be replaced by the following text:

Thanks to persons not listed as co-authors but who contributed to this work, especially the Chicago, Nigerian and Cameroonian teams.

The authors provided contributions in: conception and design, final approval, accountable for all aspects of the work.

